# Supplementary material for: Paraspeckle condensation is controlled via TDP-43 polymerization and linked to neuroprotection
Source: Nat Cell Biol. 2026 Mar 18;28(4):754–70. doi: 10.1038/s41556-026-01895-y (PMC13086584; doi:10.1038/s41556-026-01895-y)
Supplement: Supplementary file 1 — Supplementary Figs. 1–3 with legends. [file 41556_2026_1895_MOESM1_ESM.pdf]

nature cell biology

Supplementary information https://doi.org/10.1038/s41556-026-01895-y

Paraspeckle condensation is controlled via TDP-43 polymerization and linked to neuroprotection

In the format provided by the authors and unedited

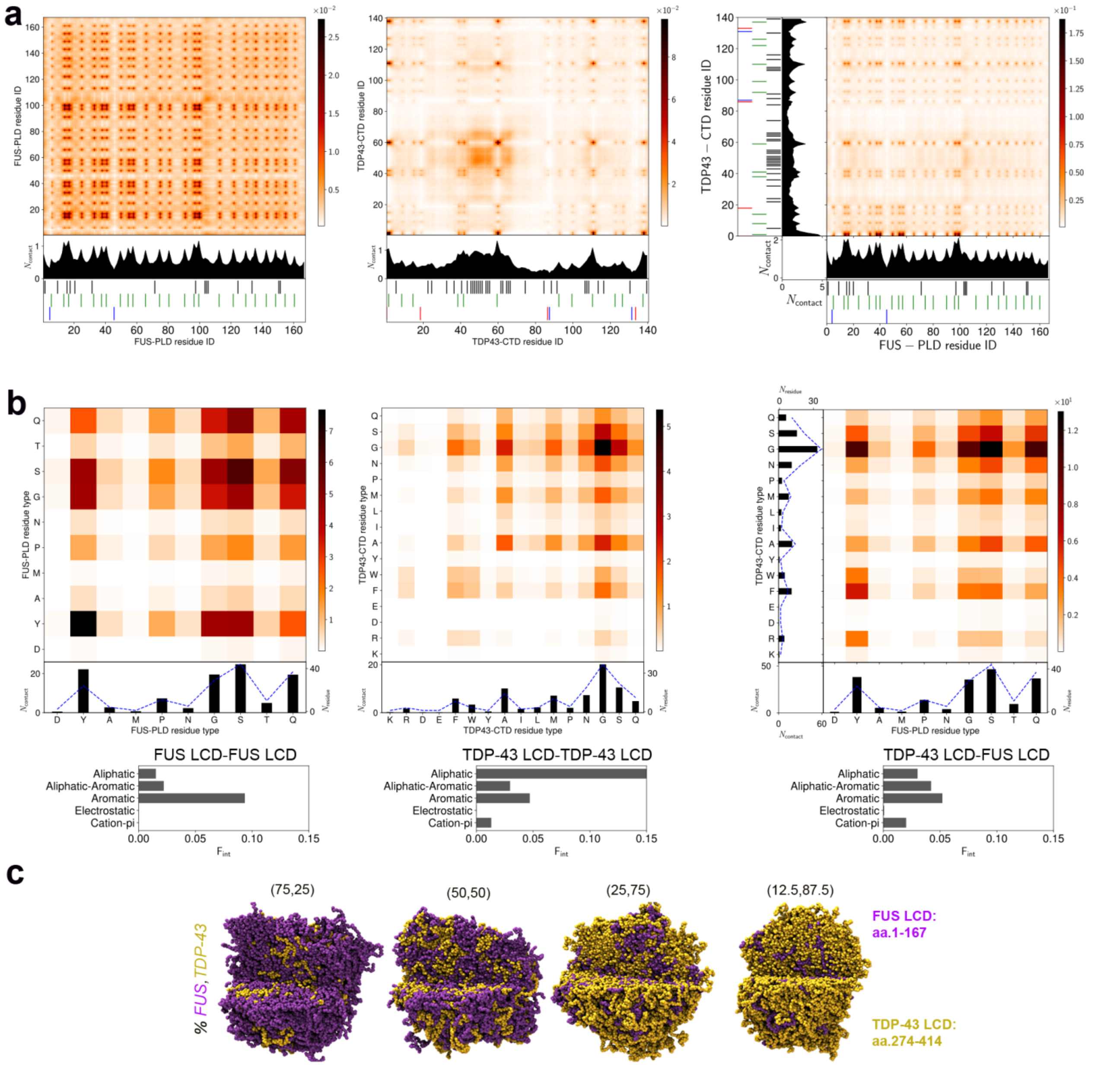

Supplementary Figure 1. Analysis of FUS LCD and TDP-43 LCD interactions by coarse-grained molecular dynamics simulations.

a) Intermolecular contacts by residue index for FUS LCD (NTD), TDP-43 LCD (CTD), and FUS LCD with TDP-43 LCD at 150 mM ion concentration and 300 K. The contacts were averaged in time and normalised by the number of molecules in the simulation. A 1D contact profile (summation of the 2D map) is included below the contact map, to show the total interactions per residue index (N contact).

b) Intermolecular contact map by residue type for FUS LCD, TDP-43 LCD, and FUS LCD with TDP-43 LCD. The contact maps in **b** are similar to the respective contact maps by residue index in **a**, but aggregated by residue type. A 1D contact profile (summation of the 2D map) is also included below (N contact), together with the abundance for the residues (N residue) shown by blue dashed lines. The fraction of interactions (F_{int}) is aggregated by type and normalised by the total number of interactions. Aromatic and aliphatic interactions denote aromatic-aromatic and aliphatic-aliphatic interactions, respectively.

c) FUS LCD - TDP-43 LCD interactions at different ratios. Simulations were all run with an amino acid concentration of 80000 μM. The total composition of a system is defined relative to 240 molecules of each protein. Intermolecular interaction summary is for FUS, TDP-43, and FUS/TDP-43 at 150 mM and 300K.

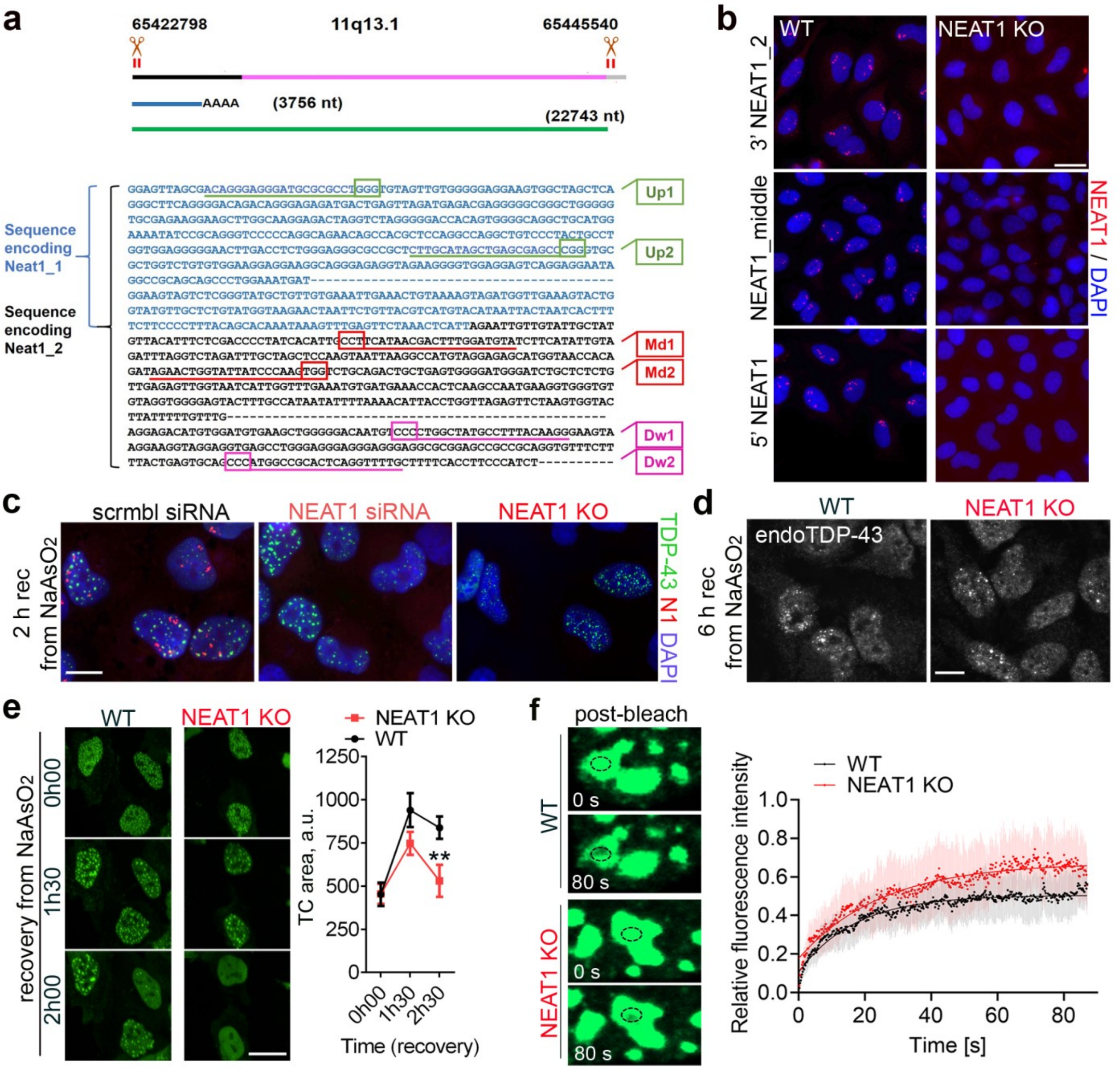

Supplementary Figure 2. Loss of NEAT1 does not prevent stress-induced nuclear TDP-43 condensate (TC) assembly but affects TC dynamics.

a) Positions of gRNAs used for NEAT1 knockout (KO). Double targeting approach (two gRNAs upstream and two gRNAs downstream of the region to be deleted) was used.

b) NEAT1 KO was confirmed by RNA-FISH using 5'-end, middle and 3'-end NEAT1 probes. Representative images are shown. Scale bar, 20 µm.

c,d) NEAT1-deficient cells are competent in TC assembly by ectopic (**c**) or endogenous (**d**) TDP-43 protein. In **c**, WT and NEAT1 KO cells were transfected to express TDP-43 GFP for 24 h, stressed with $NaAsO_2$ for 1 h, washed and analysed 2 h into the recovery (recovery stage 2/3). For NEAT1 knockdown, WT cells were co-transfected with NEAT1 or scrambled siRNA. NEAT1 (N1) RNA-FISH was used to confirm NEAT1 depletion. In **d**, TC assembly by endogenous TDP-43 was analysed 6 h into the recovery. Representative images are shown. Scale bar, 10 µm.

e) TC clearance is accelerated in NEAT1 KO cells. WT and NEAT1 KO cells expressing TDP-43 GFP were stressed with $NaAsO_2$, washed and analysed by time-lapse confocal imaging during the recovery. Time of recovery is indicated. Representative images are shown. >500 cells were analysed per condition by automated quantification, from n=3 wells, from a representative experiment. Graph shows mean±s.e.m. **p<0.01 (p=0.003), one-tailed Mann-Whitney *U* test.

f) TCs are more dynamic in NEAT1 KO cells, as demonstrated by FRAP. Data from a representative experiment are shown (n=10 cells, 3 condensates in each).

HeLa cells were used for these studies.

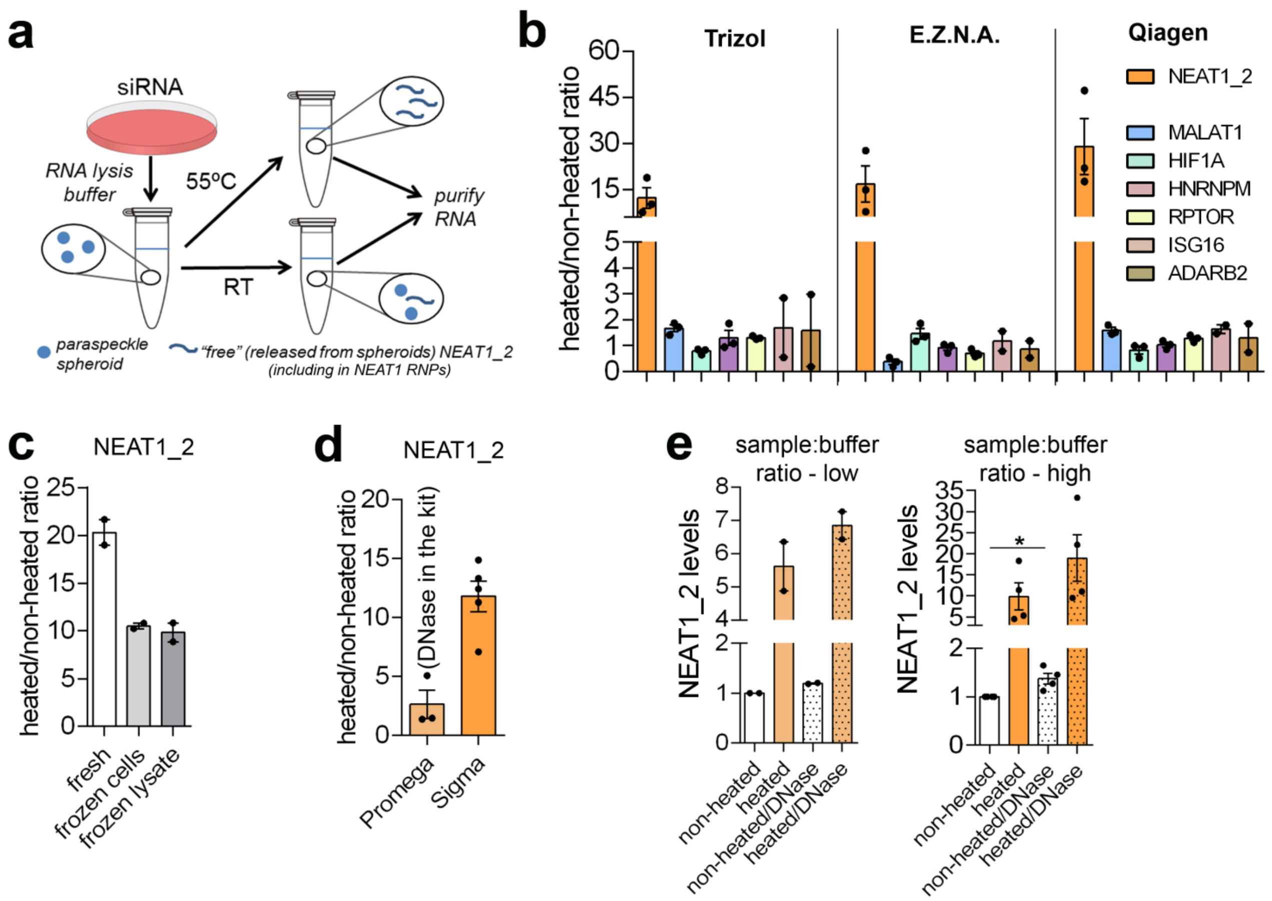

Supplementary Figure 3. NEAT1_2 semi-extractability and its modifiers.

a) NEAT1_2 semi-extractability analysis approach.

b) NEAT1_2, but not other RNAs, is semi-extractable in RNA samples purified using commercial kits. qRT-PCR analysis of a panel of RNAs using 3 commercial kits. N=2 for ISG16 and ADARB2 and N=3 for all other genes, for all kits tested.

c) Freeze-thaw treatment of cell pellets or lysed samples reduces NEAT1_2 semi-extractability. Total RNA was purified using the Sigma GenElute total RNA kit from freshly harvested cells ("fresh"), cell pellets snap-frozen at 80ºC ("frozen cells") and cells lysed in the kit's lysis buffer and subsequently snap-frozen in -80ºC ("frozen lysate"). N=2.

d) Inclusion of an on-column DNase digest step into the purification protocol reduces NEAT1_2 semi-extractability. Commercial kits that include (Promega) or not include (Sigma) DNase treatment in the standard protocol were compared. N=3 or 4.

e) NEAT1_2 is more semi-extractable in high-density (highly concentrated) samples/lysates. High and low sample/buffer ratios: $3x10^6$ and $0.75x10^6$ cells per 1 ml lysis buffer, respectively. Note that DNase treatment potentiates NEAT1_2 extraction, consistent with data in **e**. N=2 or 4, *$p<0.05$ ($p=0.0143$), one-tailed Mann-Whitney *U* test. For the DNase-free condition, DNase was heat-inactivated prior to addition to the columns. Sigma GenElute total RNA kit was used in these studies.

HeLa cells were used for these studies. All graphs show mean±s.e.m.
