# Supplementary material for: Paraspeckle condensation is controlled via TDP-43 polymerization and linked to neuroprotection
Source: Nat Cell Biol. 2026 Mar 18;28(4):754–70. doi: 10.1038/s41556-026-01895-y (PMC13086584; doi:10.1038/s41556-026-01895-y)
Supplement: Supplementary file 2 — Reporting summary [file 41556_2026_1895_MOESM2_ESM.pdf]

nature portfolio

Corresponding author(s): Tatyana Shelkovnikova

Last updated by author(s): 12 October 2025

Reporting Summary

Nature Portfolio wishes to improve the reproducibility of the work that we publish. This form provides structure for consistency and transparency in reporting. For further information on Nature Portfolio policies, see our Editorial Policies and the Editorial Policy Checklist.

Statistics

For all statistical analyses, confirm that the following items are present in the figure legend, table legend, main text, or Methods section.

n/a	Confirmed	
☐	☒	The exact sample size (*n*) for each experimental group/condition, given as a discrete number and unit of measurement
☐	☒	A statement on whether measurements were taken from distinct samples or whether the same sample was measured repeatedly
☐	☒	The statistical test(s) used AND whether they are one- or two-sided<br>*Only common tests should be described solely by name; describe more complex techniques in the Methods section.*
☒	☐	A description of all covariates tested
☐	☒	A description of any assumptions or corrections, such as tests of normality and adjustment for multiple comparisons
☐	☒	A full description of the statistical parameters including central tendency (e.g. means) or other basic estimates (e.g. regression coefficient) AND variation (e.g. standard deviation) or associated estimates of uncertainty (e.g. confidence intervals)
☐	☒	For null hypothesis testing, the test statistic (e.g. *F*, *t*, *r*) with confidence intervals, effect sizes, degrees of freedom and *P* value noted<br>*Give P values as exact values whenever suitable.*
☒	☐	For Bayesian analysis, information on the choice of priors and Markov chain Monte Carlo settings
☒	☐	For hierarchical and complex designs, identification of the appropriate level for tests and full reporting of outcomes
☒	☐	Estimates of effect sizes (e.g. Cohen's *d*, Pearson's *r*), indicating how they were calculated

Our web collection on statistics for biologists contains articles on many of the points above.

Software and code

Policy information about availability of computer code

Data collection	The following software was used for data collection: ZEN blue software (Zeiss); CellSens Dimension software (Olympus); Harmony 4.9 High-Content Imaging and Analysis Software (Revvity); Image Studio (LICORbio); NIS Elements v2.20.02 (Nikon); FIDA Software Suite (Fidabio); Pymol v.2.55.5; Image J 1.54m.
Data analysis	No original code was generated in this study.

For manuscripts utilizing custom algorithms or software that are central to the research but not yet described in published literature, software must be made available to editors and reviewers. We strongly encourage code deposition in a community repository (e.g. GitHub). See the Nature Portfolio guidelines for submitting code & software for further information.

Data

Policy information about availability of data

All manuscripts must include a data availability statement. This statement should provide the following information, where applicable:

- Accession codes, unique identifiers, or web links for publicly available datasets
- A description of any restrictions on data availability
- For clinical datasets or third party data, please ensure that the statement adheres to our policy

All data that are necessary to interpret, verify and extend the research in the article are provided in the main and extended figures, supplementary data or source files. Expression plasmids generated in the study are available from the Addgene repository: https://www.addgene.org/Tatyana_Shelkovnikova/ All other unique

study materials are available from the corresponding author; a signed Material Transfer Agreement (MTA) may be required for transfer. Source data are provided with this study. All other data supporting the findings of this study are available from the corresponding author on reasonable request.

Research involving human participants, their data, or biological material

Policy information about studies with human participants or human data. See also policy information about sex, gender (identity/presentation), and sexual orientation and race, ethnicity and racism.

Reporting on sex and gender	Not applicable
Reporting on race, ethnicity, or other socially relevant groupings	Not applicable
Population characteristics	The 6,739 individuals including 4,996 ALS patients and 1,743 controls subject to WGS and included in this study were recruited at specialised neuromuscular centres in the UK, Belgium, Germany, Ireland, Italy, Spain, Turkey, the United States and the Netherlands.
Recruitment	Patients were diagnosed with possible, probable or definite ALS according to the 1994 El-Escorial criteria. All controls were free of neuromuscular diseases and matched for age, sex and geographical location.
Ethics oversight	Project MinE was approved by the Trent Research Ethics Committee 08/H0405/60. Informed consent for genetic research was obtained from all participants.

Note that full information on the approval of the study protocol must also be provided in the manuscript.

Field-specific reporting

Please select the one below that is the best fit for your research. If you are not sure, read the appropriate sections before making your selection.

☒ Life sciences ☐ Behavioural & social sciences ☐ Ecological, evolutionary & environmental sciences

For a reference copy of the document with all sections, see nature.com/documents/nr-reporting-summary-flat.pdf

Life sciences study design

All studies must disclose on these points even when the disclosure is negative.

Sample size	RNA expression analysis (qRT-PCR): a minimum 3 biological replicates were analysed in the experiments with statistical significance analysis.<br>Cell phenotype quantification studies: experiments were repeated at least 3 times, and quantification was done typically in at least 40 cells (for transient transfection, and typically more) per condition, from 10 fields of view in a representative experiment. Exceptions: in highly homogeneous populations, where nearly all cells showed the same phenotype, cell numbers were >20. Number of intracellular condensates analysed (e.g. paraspeckles) was typically >100.<br>In vitro imaging experiments: experiments were repeated at least 3 times, and at least 4 fields of view were analysed from a representative experiment.<br>Biochemical analysis (e.g. western blot) experiments were repeated at least 3 times (N=3).<br>FRAP experiments: experiments were repeated at least 3 times, with 10 or more cells analysed in each experiment (2 or more particles in each cell).<br>The numbers and biological and technical repeats were determined for each type of experiment based on prior extensive experience in this type of studies.
Data exclusions	No data exclusions have been made, except a single outlier in the human genetic dataset analysis.
Replication	Only data where replication was achieved in at least 3 experiments were used for conclusions and included in the manuscript.
Randomization	Not relevant - the experiments did not involve randomisation.
Blinding	Investigators were blinded to the condition in experiments requiring manual quantification, where possible.

Reporting for specific materials, systems and methods

We require information from authors about some types of materials, experimental systems and methods used in many studies. Here, indicate whether each material, system or method listed is relevant to your study. If you are not sure if a list item applies to your research, read the appropriate section before selecting a response.

Materials & experimental systems

n/a	Involved in the study
☐	☒ Antibodies
☐	☒ Eukaryotic cell lines
☒	☐ Palaeontology and archaeology
☒	☐ Animals and other organisms
☒	☐ Clinical data
☒	☐ Dual use research of concern
☒	☐ Plants

Methods

n/a	Involved in the study
☒	☐ ChIP-seq
☒	☐ Flow cytometry
☒	☐ MRI-based neuroimaging

Antibodies

Antibodies used

Antibody, Company, Catalogue number, and Lot number:
FUS (rabbit polyclonal) Proteintech Cat#11570-1-AP, 00107882
FUS (mouse monoclonal) Santa Cruz Cat#sc-47711, K1915
TDP-43 (rabbit polyclonal) C-terminal Sigma Cat# T1580, 0000193756
TDP-43 (mouse monoclonal) R&D Biosystems Cat# MAB7778, CHGW0123021
TDP-43 E2G6G (rabbit monoclonal) Cell Signaling Cat# #89718, Lot 1
mCherry (rabbit polyclonal) Proteintech Cat# 26765-1-AP, 00062779
GFP (rabbit polyclonal) Proteintech Cat# 50430-2-AP, 00150738
Tuj (Alexa®488-conjugated), Abcam, Cat# ab237350
Secondary fluorescently labelled antibodies: Alexa488/546/633 Fluor anti-mouse/rabbit IgG, ThermoFisher: Rabbit 488 #A-11008, 2897813; Mouse 488 #A-11001, 2551357; Mouse 546 #A-11030, 2465085; Rabbit 546, #A11010, 2902337; Mouse 633 #A21050, 2920646.
Mouse IgG HRP Linked Whole Ab Amersham Cat# NA931, 18095933
Rabbit IgG HRP Linked Whole Ab Amersham Cat# NA934, 18225602

Primary antibodies were used at 1:1000 dilution for immunocytochemistry and western blot and at 1:5000 in ImmuCon. Secondary antibodies were used at 1:1000 dilution.

Validation

Primary antibodies were validated in the required application prior to use: by western blot (single dominant band of predicted molecular weight) and ICC (expected/known subcellular distribution); select antibodies - by siRNA knockdown (and subsequent WB and ICC).

Eukaryotic cell lines

Policy information about cell lines and Sex and Gender in Research

Cell line source(s)

Human: HeLa cells (ATCC), female, Merck, Cat# 93021013
Human: SH-SY5Y cells (ATCC), female, Merck, Cat# 94030304
Human: MCF7 cells (ECACC), female, Merck, Cat# 86012803
Human: FUS knockout SH-SY5Y cell line - the above parental line was used
Human: NEAT1 knockout HeLa cell line - the above parental line was used
Human: NEAT1_2 Δrep4 HeLa cell line - the above parental line was used
Human: 1xMS2-NEAT1_2 HeLa cell lines - the above parental line was used
Human: KOLF2.1J iPSC line, male, JAX, Cat#JIPSC001000
Human: NEAT1_2 Δrep4 iPCS line - the above parental line was used
Human: NEAT1_2 KO iPCS line - the above parental line was used

Authentication

The cell lines were procured directly from the approved repository vendor, with all the authentication documentation (e.g. STR profiling) available.

Mycoplasma contamination

Mycoplasma testing of all cell lines was performed routinely (every 2 weeks) using a sensitive luminescence kit; all cell lines stayed mycoplasma-free throughout the study.

Commonly misidentified lines
(See ICLAC register)

Such lines were not used in the study.

Plants

Seed stocks	N/A
Novel plant genotypes	N/A
Authentication	N/A
