# Supplementary material for: Paraspeckle condensation is controlled via TDP-43 polymerization and linked to neuroprotection
Source: Nat Cell Biol. 2026 Mar 18;28(4):754–70. doi: 10.1038/s41556-026-01895-y (PMC13086584; doi:10.1038/s41556-026-01895-y)

Main Fig. 1j

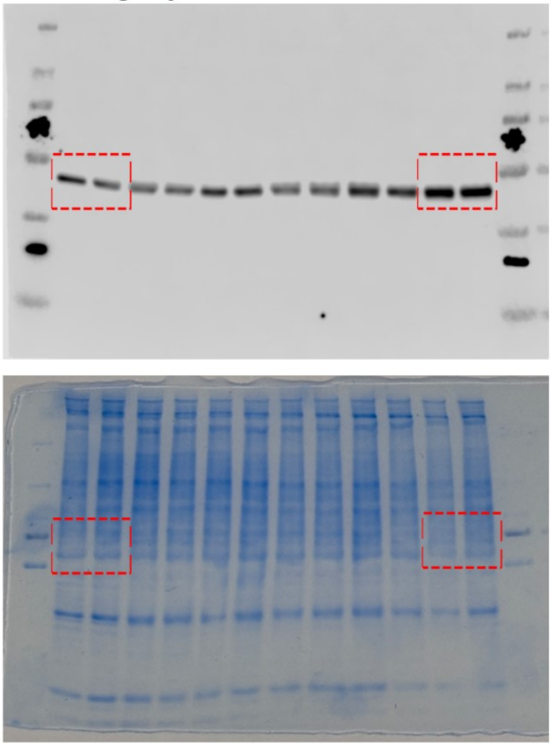

Main Fig. 4b

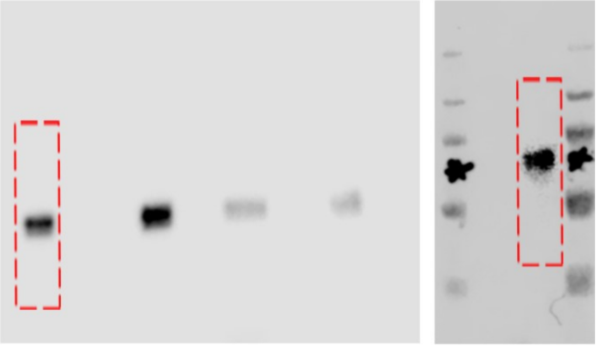

Main Fig. 4j

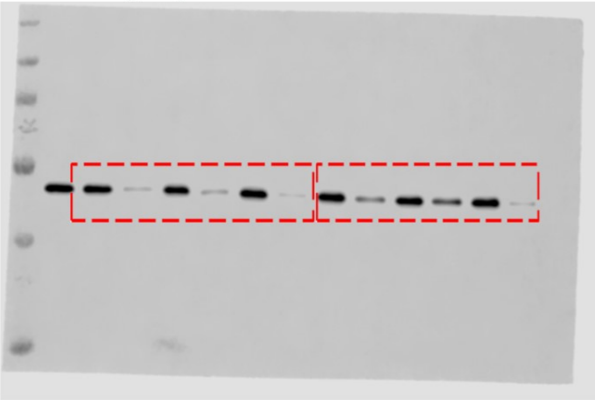

Extended Fig. 1e

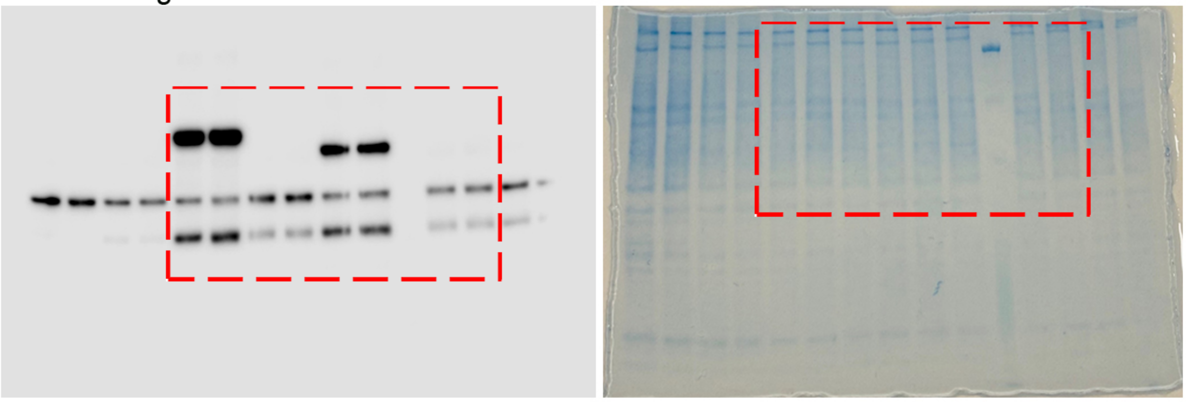

Extended Fig. 3a

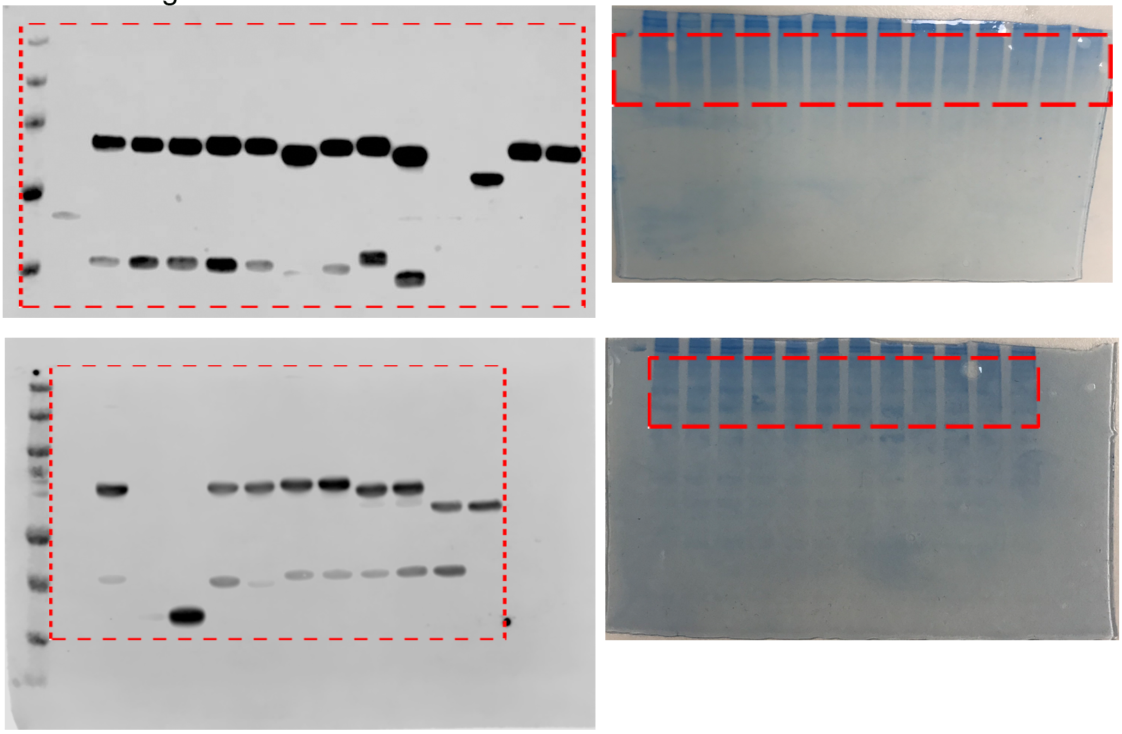

Extended Fig. 3c

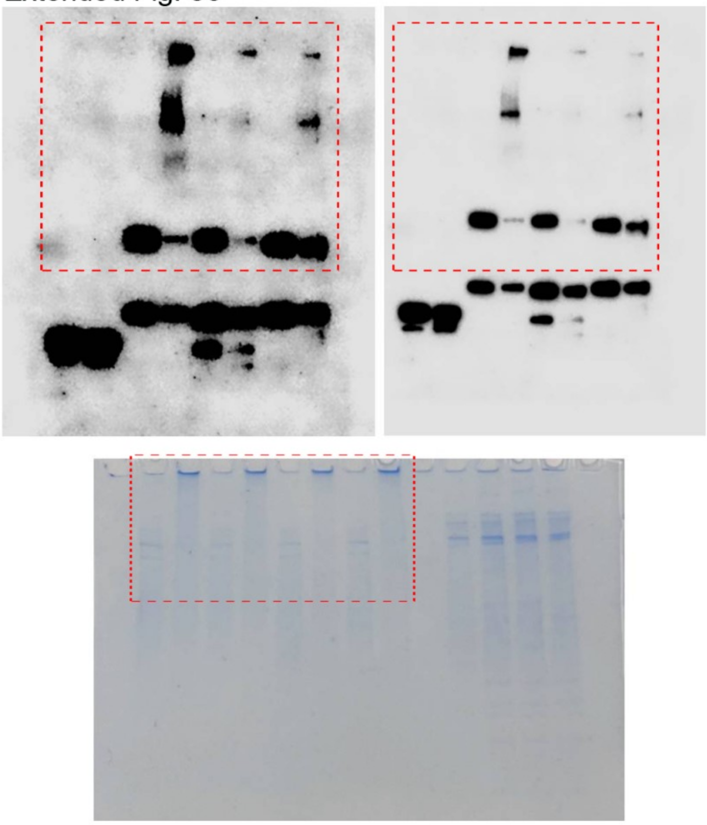

Extended Fig. 3f

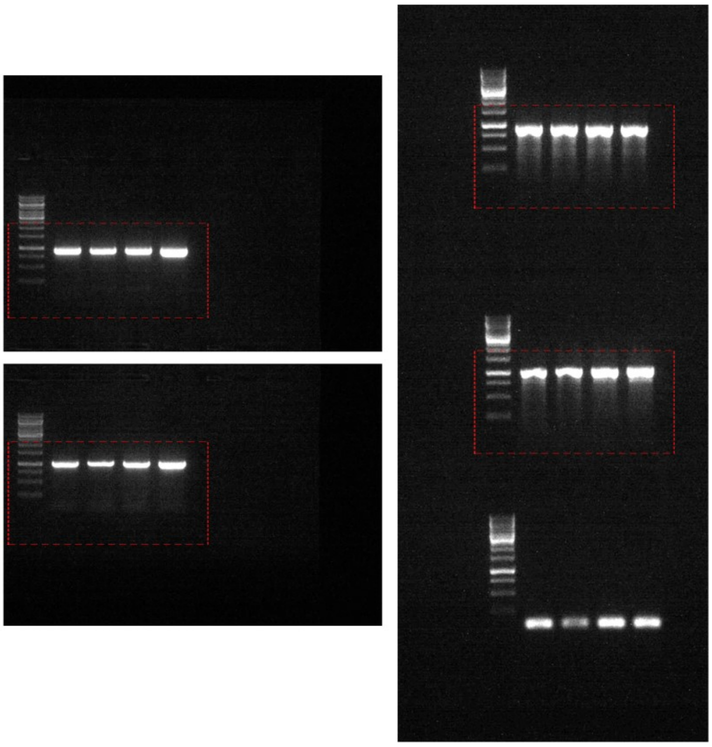

Extended Fig. 4d

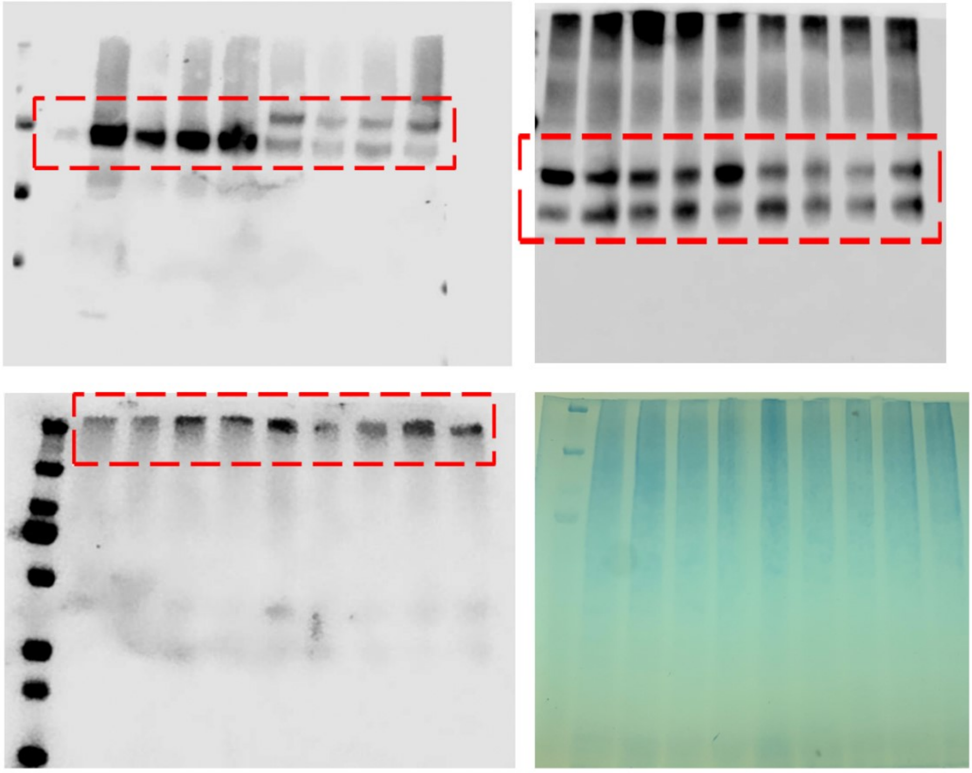

Extended Fig. 5a

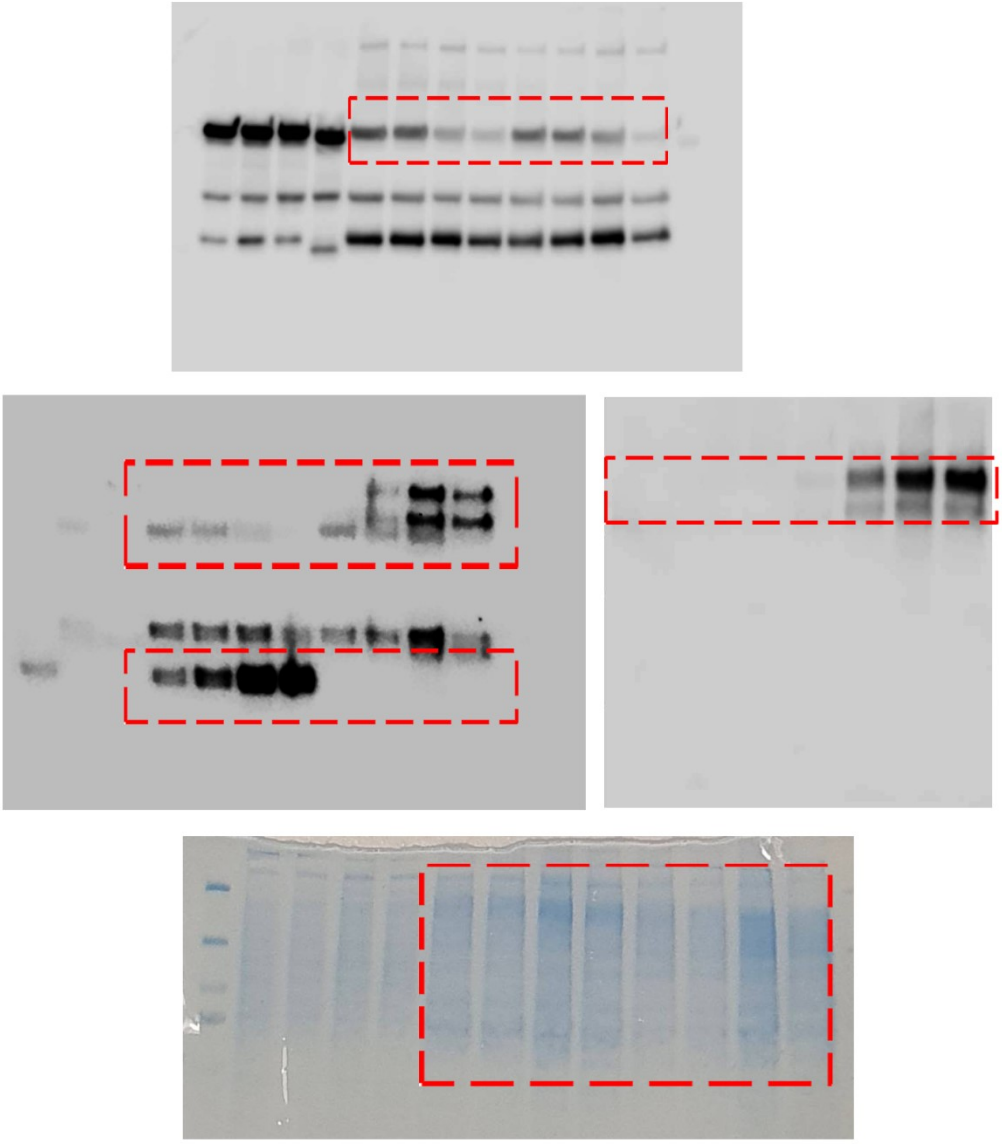

Extended Fig. 6b

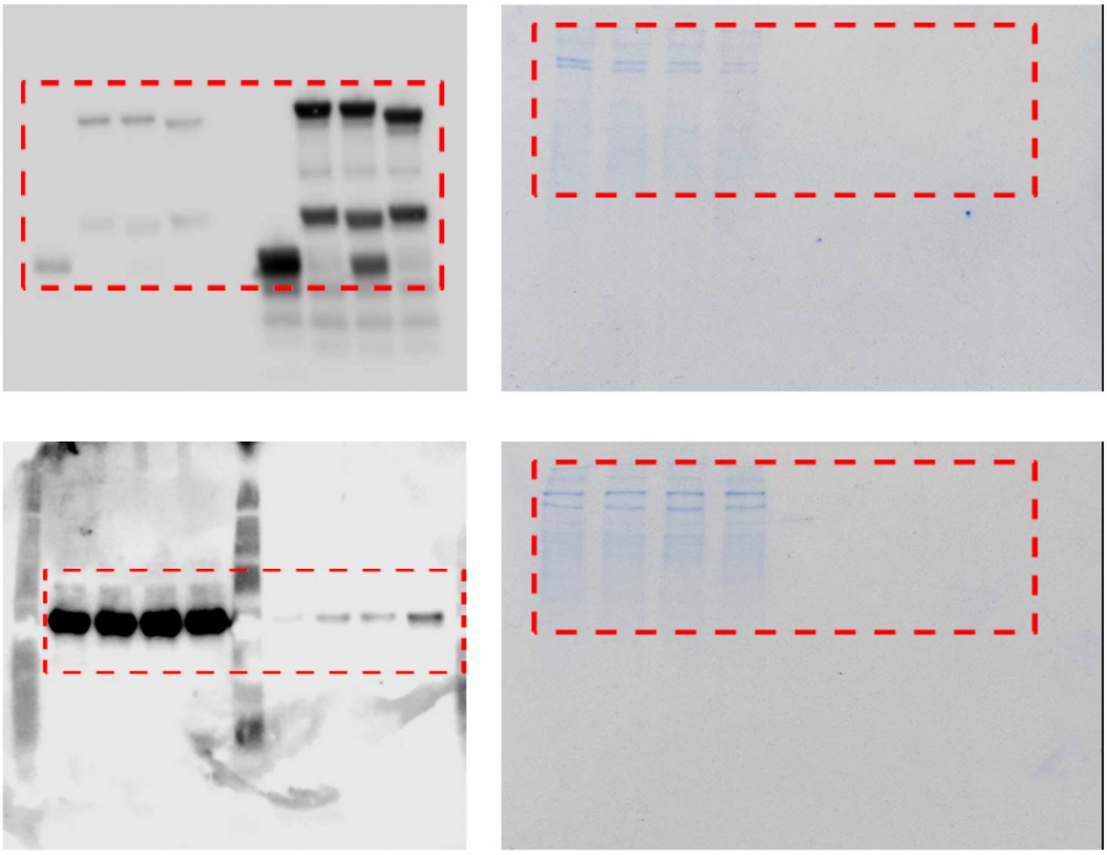

Extended Fig. 7a

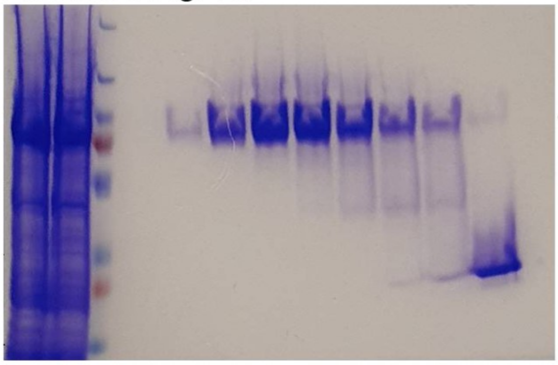

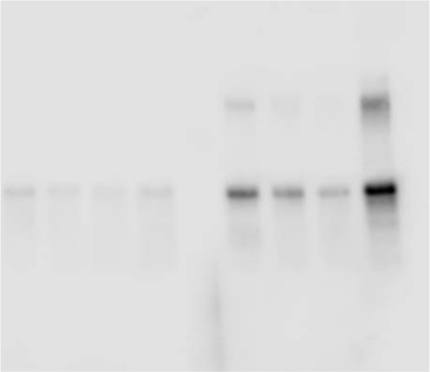

Extended Fig. 7b

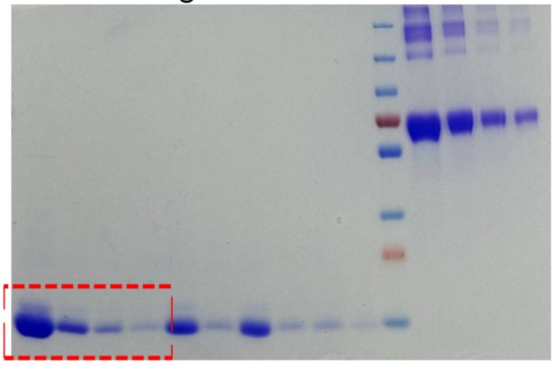

Extended Fig. 8f

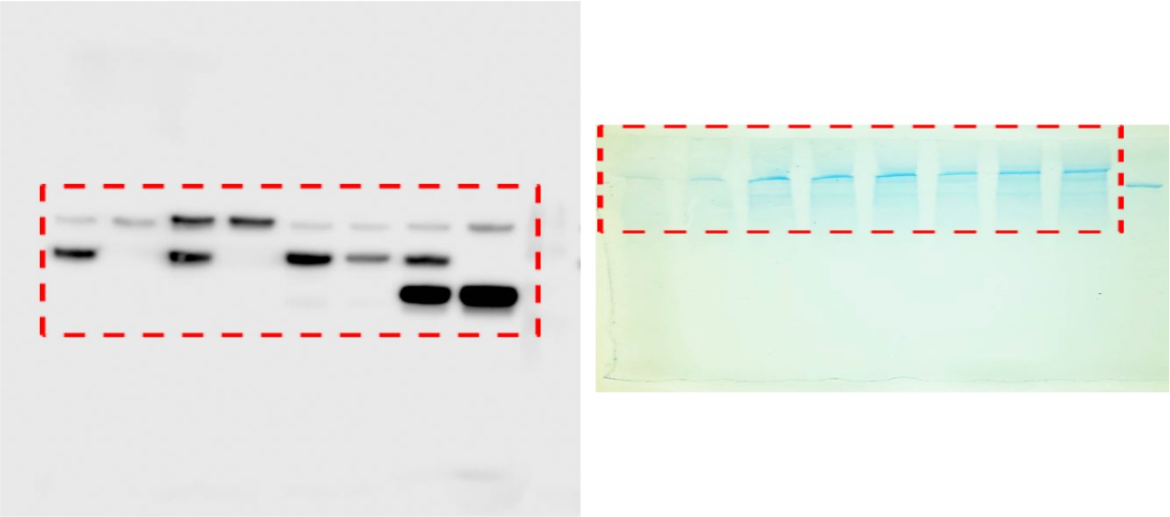

Extended Fig. 8g

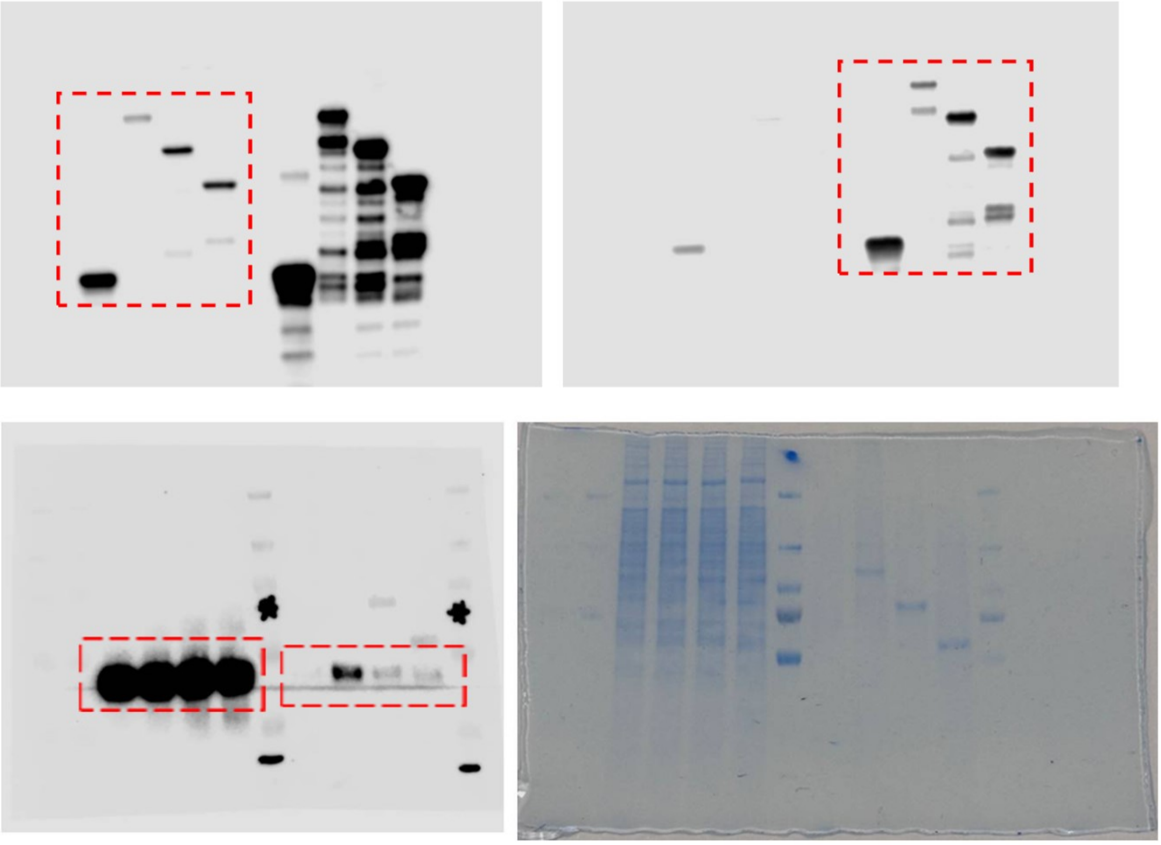

Extended Fig. 10b

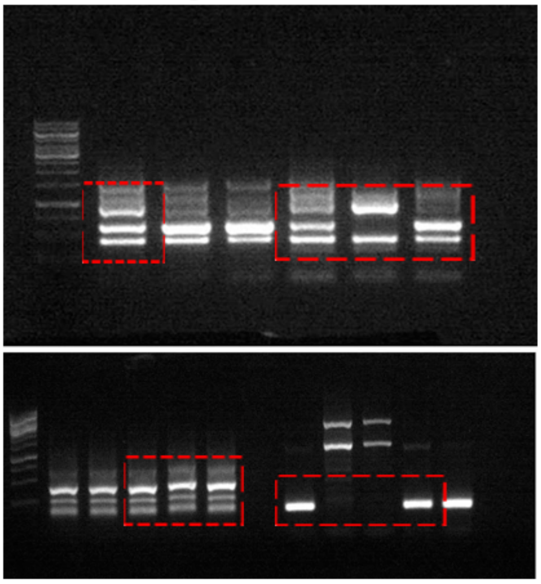

Extended Fig. 10f and g

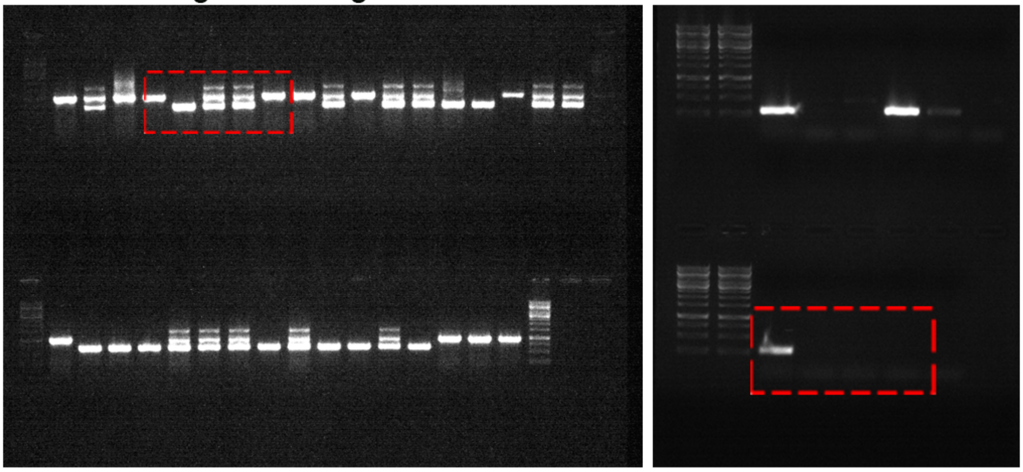

Extended Fig. 10i

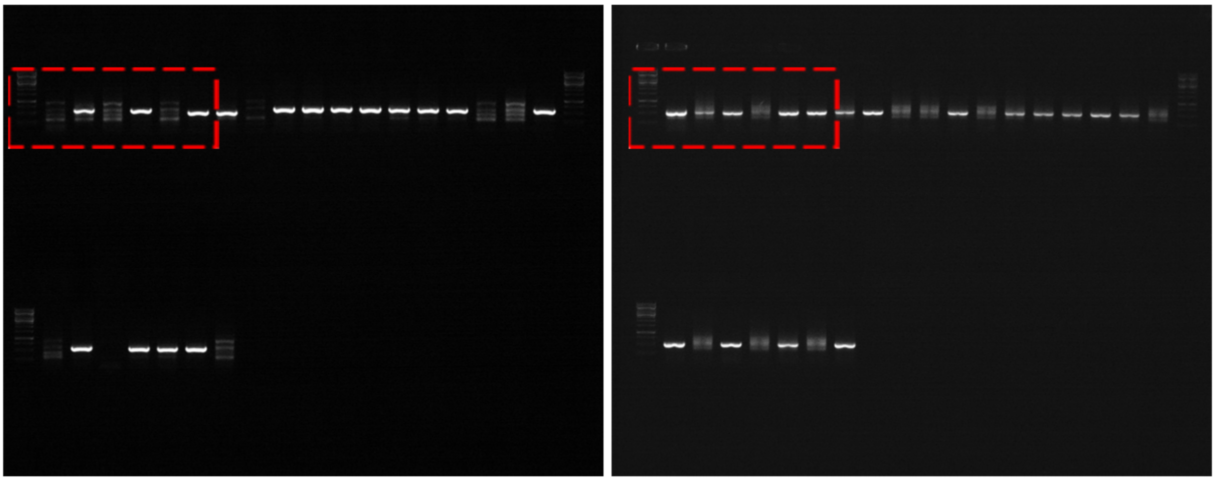

Supplement: Supplementary file 7 — Unprocessed western blots and/or gels. [file 41556_2026_1895_MOESM7_ESM.pdf]
